# How explicable are differences between reviews that appear to address a similar research question? A review of reviews of physical activity interventions

**DOI:** 10.1186/2046-4053-1-37

**Published:** 2012-08-17

**Authors:** Jenny Woodman, James Thomas, Kelly Dickson

**Affiliations:** 1 MRC Centre for Epidemiology of Child Health, UCL-Institute of Child Health, 30 Guilford Street, London, WC1N 1EH, UK; 2 EPPI-Centre, Social Science Research Unit, Institute of Education, University of London, 18 Woburn Square, London, WC1H 0NR, UK; 3 EPPI-Centre, Social Science Research Unit, Institute of Education, University of London, 18 Woburn Square, London, WC1H 0NR, UK

**Keywords:** Systematic review, Methods, Community interventions, Physical activity

## Abstract

**Background:**

Systematic reviews are promoted as being important to inform decision-making. However, when presented with a set of reviews in a complex area, how easy is it to understand how and why they may differ from one another?

**Methods:**

An analysis of eight reviews reporting evidence on effectiveness of community interventions to promote physical activity. We assessed review quality and investigated overlap of included studies, citation of relevant reviews, consistency in reporting, and reasons why specific studies may be excluded.

**Results:**

There were 28 included studies. The majority (*n* = 22; 79%) were included only in one review. There was little cross-citation between reviews (*n* = 4/28 possible citations; 14%). Where studies appeared in multiple reviews, results were consistently reported except for complex studies with multiple publications. Review conclusions were similar. For most reviews (*n* = 6/8; 75%), we could explain why primary data were not included; this was usually due to the scope of the reviews. Most reviews tended to be narrow in focus, making it difficult to gain an understanding of the field as a whole.

**Conclusions:**

In areas where evaluating impact is known to be difficult, review findings often relate to uncertainty of data and methodologies, rather than providing substantive findings for policy and practice. Systematic ‘maps’ of research can help identify where existing research is robust enough for multiple in-depth syntheses and also show where new reviews are needed. To ensure quality and fidelity, review authors should systematically search for all publications from complex studies. Other relevant reviews should be searched for and cited to facilitate knowledge-building.

## Background

One of the principles underpinning evidence informed policy and practice is that of knowledge accumulation: that we do the most good, and avoid harms, by basing our decisions on systematic reviews of high quality research [1]. Systematic reviews can synthesize a large amount of sometimes conflicting evidence and can therefore be a potentially important influence on practitioner and policy-makers’ decisions [2].

However, how suitable are systematic reviews for informing decision-making when using reviews that were not commissioned for that specific decision? The applicability of review findings has been called into question recently, with some reviews being criticized for lacking the context-specific detail that is essential to translate their findings to specific practical situations [3]. Equally important is the question of whether systematic reviews can be relied upon genuinely to reflect the state of the evidence base. To do this they must: (1) ensure that all relevant studies are identified through the use of exhaustive search strategies; and (2) ensure that their conclusions are based on reliable studies.

In this paper we examine eight reviews of community interventions to promote physical activity in order to investigate the issue of comprehensiveness and reliability in reviews and to consider the problem of applicability; if we were a practitioner wishing to use these reviews to inform our practice, how confident can we be that our decision would be based on all the available evidence and that the conclusions drawn were reliable? And, while the reviews we may seek to draw upon will all appear to address similar questions, are we able to mediate differences between them?

In essence, we placed ourselves in the hypothetical position of wanting to identify evidence about ‘what community interventions work’ to promote physical activity among children to inform our decision-making. Using a systematic ‘map’ of reviews we selected a set of reviews that are ostensibly all about the same broad issue – that of community interventions to promote physical activity. Our confidence in the evidence base as portrayed by the reviews would be increased if we could see how they each contributed to an overall understanding of the field; and if reviews addressing the same question identified the same studies and treated them in the same way. If they did not, we might worry that other, equally relevant, studies were missing too, and without considerable effort on our part, we would have no way of mediating between conflicting findings.

We wished to explore any differences between reviews in terms of the studies they included. While there may be legitimate reasons for reviews on the same subject not containing the same studies (for example, differences in scope or population) it may be that it is difficult to understand the inclusion or exclusion of certain studies purely in terms of the scope of reviews; where there are differences between reviews that cannot be explained by their having different scopes or purposes, these differences might be explained in terms of differential review quality. In addition, we would hope that reviews which included the same studies would report the same results from those studies, draw the same conclusions, given their similar scope, and reach similar conclusions about the effectiveness of community interventions for physical activity.

One of the justifications for systematic reviews is encapsulated by the concept ‘knowledge accumulation’; that new research should build on previous work and say how it contributes to existing knowledge. Additionally, locating new systematic reviews in the context of other reviews should facilitate the process of piecing together knowledge from multiple reviews in order to inform practical decisions. To see how far the eight reviews facilitated this, we investigated how far the reviews cited one another, since inter-citation may be taken as evidence that reviewers were both aware of previous work and sought explicitly to advance the state of knowledge in their area.

Since it is not always clear whether a review is systematic or non-systematic, and given that literature reviews are often commissioned to inform decisions, we included all types of literature in this area (not just systematic reviews) and assessed the relationship between review methods and included data, reporting, and conclusions.

Our research questions were:

1. To what extent do reviews answering a similar research question include the same primary studies?

2. Where reviews do not contain the same studies, is this explicable in terms of differences in their scope?

3. How similarly do reviews answering a similar research question report the results of the primary studies they have in common?

4. To what extent do reviews answering a similar research question draw the same conclusions?

5. To what extent do reviews answering a similar research question cite other reviews on the same topic?

6. Does the methodological quality of reviews answering a similar research question help us to understand any differences between included studies in terms of results and conclusions**?**

## Methods

### Identifying reviews which answer similar research questions

In 2008 we published a systematic map of reviews on ‘Social and environmental interventions to reduce childhood obesity’ which included 33 reviews about the impact of upstream or ‘social and environmental’ interventions on eating, physical activity, sedentary behavior, and/or associated attitudes [4]. This map included reviews about physical activity (or sedentary behavior), healthy eating (or weight management) with an OECD country focus, and which included children in their topics of focus. In order to investigate study overlap between reviews, we needed a sufficiently large sample of reviews that were as homogenous as possible in terms of their topic areas. We therefore used a subset of the reviews in the above ‘map’ to be the focus of our investigation. This subset of reviews investigated the effectiveness of community interventions to promote physical activity (either alone or in combination with healthy eating). There were 16 such reviews in the above ‘map’ but in order to maximize comparability of research question and scope of the reviews, we excluded reviews which:

1. only had very little effectiveness data (for example, were primarily a description of funded interventions);

2. had inclusion criteria that restricted the population in terms of ethnicity, race, or age (for example, only included studies about Aboriginal/Torres Strait Islander people);

3. did not draw any conclusions about physical activity.

On this basis, we excluded eight reviews [5-11]. We tabulated the inclusion criteria of the remaining eight reviews (Table 1) [12-19] and judged that they were similar enough in scope to be compared. Although two reviews [14,15] had been updated since our searches (2007–2008), we based our analyses on the original reviews included in our map.

**Table 1:** **Characteristics of the reviews which met our inclusion criteria (*n*= 8)**

**Review authors and date**	**Stated aim of review**	**Dates covered**	**Publication type/language**	**Population**	**Intervention type**	**Setting**	**Study design**	**Outcome measured**	**AMSTAR assessment**^**a**^
Dobbins & Beyers, 1999 [12]	‘To assess, analyze, and draw conclusions about the effects of heart health community-based initiatives’	1994-1998	No restriction	No restriction	Community heart health projects with community involvement in delivery	Community	Randomized and non-randomized control trials	No restriction	1: Y
									2: Y
									3: Y
									4: N
									5: Y
									6: Y
									7: Y
									8: Y
									9: Y
									10: N
									11: Y
									‘Systematic’
Fogelholm & Lahti-Koski, 2002 [16]	‘Summarize the results of large community interventions for prevention of cardiovascular diseases, with dietary changes and increased physical activity as target behaviors, and change in obesity as one outcome variable”	1990-2002	No restriction	No restriction	Any physical activity intervention with a PA component	Community	No restriction	Body weight, BMI, obesity	1: CA
									2: N
									3: N
									4: N
									5: Y
									6: Y
									7: N
									8: N
									9: Y
									10: N
									11: N
									‘Non-systematic’
Jackson *et al*. 2005a [14]	‘To determine the effects of interventions implemented through sporting organizations to increase (active and non-active) participation in organized sport.’	No restriction −2004	No restriction	No restriction	Any intervention implemented through sporting organizations to increase sport	No restriction	Non-randomized and randomized controlled trials.	Participation in sport	1: Y
									2: Y
									3: Y
									4: Y
									5: NA^b^
									6: NA
									7: NA
									8: NA
									9: NA
									10: Y
									11: Y
									‘Systematic’
Jackson *et al*. 2005b [15]	‘To determine if policy interventions implemented through sporting organizations instigate and sustain healthy behavior change within the sport setting’	No restriction −2004	No restriction	No restriction	Policy intervention implemented through sporting organizations intended to instigate and/or sustain healthy behavior change	No restriction	Non-randomized and randomized controlled trials.	Behavior change, intentions, attitudes, knowledge, changes in policy.	1: Y
									2: Y
									3: Y
									4: Y
									5: NA^b^
									6: NA
									7: NA
									8: NA
									9: NA
									10: Y
									11: Y
									‘Systematic’
King, 1998 [17]	‘To describe the conceptual and strategic differences between community-level and individual-level approaches to activity promotion, and to highlight some examples of promising community intervention programs that have been evaluated systematically.’	Unclear	Unclear	Unclear	Any community intervention to promote PA (implicit)	Unclear	Unclear	Unclear (behavior change most frequently reported)	1: CA
									2: CA
									3: CA
									4: CA
									5: N
									6: N
									7: N
									8: N
									9: Y
									10: N
									11: N
									‘Non-systematic’
Murphy and Bauman, 2007 [13]	‘Large-scale, one-off sporting or physical activity events are often thought to impact population PA levels. this article reviews the evidence and explores the nature of the effect’	No restriction −2005	No restriction	No restriction	Elite or mass sporting event; major population level health promotion events	Community	No restriction	No restriction	1: CA
									2: CA
									3: N
									4: N
									5: N
									6: N
									7: N
									8: N
									9: CA
									10: N
									11: N
									‘Non-systematic’
Pate, 2000 [18]	To summarize literature on “community-based promotion of physical activity and proper diet among children and youth” (rationale, characteristics and impact)	Not clear	Not clear	Youth (implicit in title)	Interventions based in ‘communities’	Unclear	Unclear	Unclear	1: CA
									2: CA
									3: CA
									4: CA
									5: N
									6: N
									7: N
									8: N
									9: Y
									10: N
									11: N
									‘Non-systematic’
Sharpe, 2003 [19]	Implicit: to review the effectiveness of interventions to promote physical activity in community settings	Unclear	Unclear	Unclear	Unclear	Unclear	Community-based	Unclear	1: N
									2: CA
									3: CA
									4: CA
									5: N
									6: N
									7: N
									8: N
									9: Y
									10: N
									11: N
									‘Non-systematic’

^a^We used the AMSTAR tool (Development of AMSTAR: a measurement tool to assess the methodological quality of systematic reviews. Shea BJ, Grimshaw JM, Wells GA, *et al*. *BMC Med Res Methodol* 2007, **7:**10) to score the reviews as follows: 1, Was an ‘*a priori*’ design provided? 2, Was there duplicate study selection and data extraction? 3, Was a comprehensive literature search performed (please see text for definition)? 4, Was the status of publication (that is, grey literature) used as an inclusion criterion? 5, Was a list of studies (included and excluded) provided? 6, Were the characteristics of the included studies provided? 7, Was the scientific quality of the included studies assessed and documented? 8, Was the scientific quality of the included studies used appropriately in formulating conclusions? 9, Were the methods used to combine the findings of studies appropriate? 10, Was the likelihood of publication bias assessed? 11, Was conflict of interest reported? Answers: Y, Yes; N, No; CA, Can’t answer; NA, Not applicable.

^b^Although these reviews did not contain any included primary studies at all, the review authors stated that they had intended to carry out a formalized data extraction process and quality assurance measures.

### Methodological quality of the included reviews

As discussed in the background, systematic reviews are promoted as an important means of ensuring decisions are informed by reliable research evidence. Unfortunately, while some reviews may describe themselves as ‘systematic’ they may not be; likewise some reviews are systematic without necessarily being described as such. We therefore assessed the quality of the reviews using the AMSTAR quality assessment tool [20] to assess the degree to which they met accepted standards for being systematic reviews (broadly examining their reporting of their inclusion (and/or exclusion) criteria; their search strategy; synthesis methods; quality assessment; details reported about included studies; and quality assurance measures (that is, screening and/or data extraction and/or quality assessment of studies completed independently by two reviewers (at least in part) and differences resolved) (Table 1). This tool was developed as a result of a systematic survey of other review quality assessment tools and a consultation exercise; it therefore identifies what are widely held to be the most important characteristics of systematic reviews. We classified reviews which have clear inclusion criteria, an adequate search strategy and quality appraisal of included studies as ‘systematic’. We included non-systematic reviews as they are often used for the same purposes as systematic reviews, and are frequently commissioned to inform policy.

Given the challenges of locating data for public health interventions [21,22], we went beyond the AMSTAR criteria and only judged a search strategy to be adequate if the authors reported all of the following: searching more than two databases using both free text and thesaurus terms; searching at least one topic specific database or journal (such as those relating to physical activity, obesity, eating or food, or public health (the scope of the original map)); and using at least one non-database search source (internet searching, website searching, contacting experts, checking reference lists, or hand-searching key-journals). Where there was no mention of the quality indicator or where it was unclear, we assumed the quality indicators were not present.

### Identifying the studies included in the reviews

We compiled a list of all the studies that were included in the above reviews. We determined whether a study had been ‘included’ in a review by assessing whether it had its findings about the effectiveness of a community intervention reported by the review authors. We defined ‘findings about effectiveness’ as being any report of the impact of a social and environmental change or any report of an observational comparison between populations with and without a specific social and environmental factor (for example, access to walking paths). We were broad in our interpretation of ‘social and environmental’ and only excluded evaluations of purely educational interventions delivered exclusively in the workplace or classroom. Outcomes relevant to ‘physical activity’ were defined as any measure of activity, sedentary behavior, knowledge, or beliefs, or body-weight, BMI, or energy intake, following an intervention with a physical activity component.

### Analysis

We entered each included study onto our review management software EPPI-Reviewer [23] and coded the studies according to: (1) the reviews in which they were included; and (2) whether there was an obvious reason for the study’s exclusion from certain reviews, based on information available from the inclusion criteria of each review and the abstract of the included studies. In two cases, the abstract was not available (one study was very old [24] and the other was a conference abstract without any details except the title [25]). We excluded these two studies from the analyses that relied on the abstract. Researcher judgment was needed to determine whether there was a likely reason for exclusion, especially where inclusion criteria were not clearly reported. Despite overlap in scope, the reviews answered different research questions (Table 1). With this in mind, we only classified the reasons for non-included primary studies as ‘unclear’ (Table 2) if we could not discern any reason at all, based on their date, scope, and inclusion criteria, why they were not included in the review. In addition, and based on a detailed reading of the full text, we described how each review reported the results of the included studies and summarized the conclusions that each review drew about the effectiveness of community interventions for promoting or increasing physical activity. Finally, we checked the reference lists of each review to establish the frequency with which the reviews cited other relevant reviews. As manuscripts are submitted many months before publication, we judged that when publication dates were within 1 year of each other, reviews were not necessarily able to cite one other.

**Table 2:** The reasons that we deduced why studies may have been excluded from each review

**Review**	**Included**^**a**^	**Publication date**	**Population outside scope of review**	**Intervention outside scope of review**	**Outcomes outside scope of review**	**Design outside scope of review**	**Reason for exclusion is unclear**
Dobbins and Beyers, 1999	6	13	0	13	0	8	0
Fogelholm and Lahti Koski, 2002	4	6	0	3	18	1	2
Jackson, 2005a	0	2	0	25	24	16	0
Jackson, 2005b	0	2	0	25	6	16	0
King, 1998	7	6	0	1	0	0	12
Pate *et al.*, 2000	3	3	20	3	0	1	0
Murphy and Bauman, 2007	3	0	0	22	0	0	2
Sharpe, 2003	14	2	2	0	0	1	7

^a^This analysis is based on 26 studies: it excludes the two studies without available abstracts online.

Categories are non-exclusive: there can be more than one reason why a primary study was not included in a review.

Multiple publications arising from one study were analyzed as a group (that is, our unit of analysis was the included study rather than publication). We found that three studies that had generated multiple publications were included in the eight reviews (Table 3).

**Table 3:** Studies with multiple included publications (the first publication in the list is the one that has been used to reference the study in the text and tables above)

**Study**	**Publications**
Minnesota Heart Health Program	Blake *et al*., 1987 [26]
	Jeffery *et al.*, 1995 [27]
	Flora *et al.*, 1993 [28]
	Kelder *et al.*, 1995 [29]
	Kelder *et al.*, 1993 [30]
	Luepker *et al.*, 1994 [31]
	Mittelmark *et al.*, 1986 [32]
	Murray *et al.*, 1990 [33]
	Perry *et al.*, 1989 [34]
Stanford Five City Project	Farquhar *et al.*, 1990 [35]
	Flora *et al.*, 1993 [28]
	Fortmann *et al.*, 1990 [36]
	Taylor *et al.*, 1991 [37]
	Young, 1996 [38]
Welsh heart project	Tudor-Smith, 1998 [39]
	Nutbeam and Catford [40]

This is a list of all publications for each study that are cited by the included reviews. It does not represent a complete list of all publications from each study.

### Quality assurance

Data about, review quality, research question, and scope (Tables 1 and 4) were extracted as part of the original project [4]. These data were independently extracted by two researchers and discrepancies resolved by recourse to the original publications or, in some cases, to a third reviewer. Identification of ‘included’ studies in the eight reviews was also carried out independently by two reviewers and differences resolved by discussion and consensus. All other analyses were conducted by one reviewer with quality assurance checks conducted by a second reviewer on a subset of data.

**Table 4:** Overlap between primary studies included in reviews

**Number of times each study included**^**a**^	**Total**	**Citation for studies**
Included in 1 review	22	[24,25,41-60]
Included in 2 reviews	4	[39,61-63]
Included in 3 reviews	0	
Included in 4 reviews	0	
Included in 5 reviews	2	[26,35]
Total studies included in any review	28^b^	

^a^The unit of analysis is the study (not publication). Where a study has multiple publications and review authors have included at least one of the publications, we have judged this study to be ‘included’ (Table 3).

^b^This table is based on 28 primary studies, including the two without an available abstract.

## Results

A total of 28 primary studies in the eight reviews met our criteria for being ‘included studies’ [24-26,35,39,41-63]. Twenty-six of these studies (93%) had an abstract available. In many cases, especially with less high-quality reviews, it was difficult to judge which studies were ‘included’ (author using result used to answer questions about effectiveness) and which studies were referenced for another reason.

### To what extent do reviews answering a similar research question include the same primary studies?

There was little overlap between data included in the eight reviews: the majority of primary studies (*n* = 22/28; 79%) were only included in one review; four studies were included in two reviews and two of the studies that had generated multiple publications were included in five reviews (Table 4). Of the six studies which were included in multiple reviews, four [39,61-63] were included in two reviews and two were included in five reviews [26,35].

### Where reviews do not contain the same studies, can we explain why not?

For most of the 26 included studies with an available abstract, it was possible to justify why primary studies had been excluded from each review, although this involved a high degree of reviewer deduction (Table 2). Systematic reviews had fewer inexplicable exclusions of studies: it was possible to explain the absence of primary studies in the three systematic reviews. The reason for exclusion was usually research design of the primary data (some reviews specified controlled trials, of which there are few in this field) or outcome (Table 2).

As we could usually justify why primary studies were not included in reviews, the limited overlap between included primary studies might also be due to slight variations in scope and inclusion criteria (Table 1) rather than only to inadequacy of search strategies.

### How similarly do reviews answering a similar research question report the results of the primary studies they have in common?

We were able to analyze similarity in reporting of primary study results for six studies which were included multiple times in five reviews (Table 5). Results were reported similarly by different review authors for the three studies which generated only one publication. However, for the remaining three studies (Welsh heart project, Minnesota Heart Health Programmed, and Stanford 5 City; Table 5), there were discrepancies between results reported by different review authors in terms of effectiveness data, subgroup analyses, and emphasis. These studies were conducted over a longer time period, with staged and multiple evaluations and, in one case, adaptation of intervention for subgroups. None of these three reviews referenced the same combination of publications generated by the two studies with multiple publications (Table 5).

**Table 5:** How the results about physical activity from the seven studies that were included in more than one review were reported in each review

**Primary study**	**Dobbins and Beyers, 1999[**[12]**]**	**Fogelholm & Lahti-Koski, 2002[**[16]**]**	**King, 1998[**[17]**]**	**Pate, 2000[**[18]**]**	**Sharpe, 2003[**[16]**]**	**Study results reported similarly by reviews?**
Brownson *et al.*, 1996 [61]	‘No statistically significant treatment effects’	‘Did not observe any significant intervention effects on physical activity […] thought there was trend towards increased physical activity in the intervention areas’	-		-	Same
Welsh heart project (Table 3)	‘Statistically significant effect in favor of the control group’ [39,40]	‘Did not observe any significant intervention effects on physical activity’ [39]	-		-	Different
Brownell *et al.*, 1980 [62]	-	-	‘Demonstrated that placing simple signs at choice points in public places […] could have a positive impact on stair use’		‘Posting signs to encourage stair use instead of the elevator, have resulted in increases of 5-18% while the sign was posted’	Same
Heirich *et al.*, 1993 [63]	-	-	‘Employees at sites that offered [intervention] reported exercising at least three times per week, compared to only about one- third of employees at the control site”		‘Some worksite programs have shown at least short-term effectiveness in increasing employees’ physical activity levels […]’	Same
Minnesota Heart Health Program (Table 3)	‘No statistically significant treatment effects’; Author referenced two publications [31,61]	‘The residents of the intervention communities of the Minnesota Heart Health Study were somewhat more physically active (self-reported) at the end of the follow-up. The increased physical activity was apparently due to an increase in activities with a low intensity’ For a special school-based element of the study ‘Girls in the intervention communities reported significantly greater amounts of exercise than girls in control communities. Boys showed a similar tendency, but the difference […] was smaller.”Author referenced three publications [29-31]	‘Some evidence that regular physical activity increased in experimental communities relative to control communities’ And ‘mass media approaches were most successful in heightening physical activity-related awareness and knowledge whereas setting-specific programs strategies that occurred over a period of time, such as those conducted in schools and worksites, were more cost- effective in increasing actual levels of physical activity participation’; Author referenced two publications [26,31]	‘Physical activity levels throughout most of the follow up period were significantly higher in the intervention community for females […] The class of 89 study could not distinguish between community and classroom effects but modest nature of the school-based activities suggests that the community based activities played an important role in the generally positive outcomes.’ Author referenced four publications [29-31,34]	‘Cohort data […] revealed an increase in physical activity in all of the communities, with the intervention communities slightly exceeding the comparison communities at the last follow-up survey’ and ’the exposure data suggest that the Minnesota Heart Health Program may not have added a great deal to the level of risk reduction activity that would have been expected without the program’; Author also reported an intervention effect for girls in the school based sub-study Author referenced one publication [31]	Different
Stanford 5 city (Table 3	‘One project reported a statistically significant treatment	‘In the Stanford Five- City Project, the intervention had a positive effect on physical activity in the independent, cross-sectional samples, but not in the cohort survey’; Author referenced two publications [36,37]	‘Some positive, albeit modest, treatment effects were found after 6 years of intervention in the physical activity area relative to the control communities’; Author referenced two publications [28,38]	‘They clearly represent the feasibility of a community based approach to the promotion of healthy eating and physical activity’; Author referenced one publication [35]	‘The educational intervention had little, if any, impact on physical activity’; Author referenced one publication [38]	Different

### To what extent do reviews answering a similar research question draw the same conclusions?

Despite the low levels of overlap of included studies in the eight reviews, the conclusions of the reviews were similar (Table 6). All review authors made cautious claims about the effectiveness of interventions in this field for increasing physical activity behavior. All reviews except for one concluded that there was limited or no evidence of effectiveness for increasing physical activity. This one review concluded that there was evidence of effectiveness in all studies but that the size of the impact was very modest [16]. Where authors discussed subgroup effects it was either to highlight a need for evidence in this area or to suggest that targeting interventions was likely to be a promising avenue for future interventions [12,16]. Five authors drew conclusions specifically relating to the quality and methods of the evidence. Four of these authors reported that good quality evidence was limited or lacking [13-15,18]. Additionally, Dobbins and Beyers suggested that there was good quality but very complex evidence [12]. The three authors who gave clear explanations of their findings [12,13,16] suggested that a lack of strong evidence for the positive impact of community interventions for physical activity might be at least partly due to difficulties in measuring impact and/or design problems such as small sample size. All authors concluded that we should not abandon community interventions to increase physical activity. Instead, they recommended that more research was needed and most gave specific recommendations.

**Table 6:** Conclusions from each review about physical activity

**Review**	**Conclusions about overall effectiveness**	**Conclusions regarding effectiveness among any subgroups**	**Conclusions about the quality of evidence contained in each review**	**Explanation of the findings in each review**	**Conclusions about the future directions of the field**
Dobbins and Beyers, 1999 [12]	‘Heart health interventions not likely to produce statistically significant effects on increasing the percentage of the population who are regularly physically active, or in decreasing the percentage of the population who are physically inactive’	We do not know if heart health interventions had an impact on specific groups of people. There is some to suggest effectiveness for high-risk populations	The community-based heart health literature is methodologically strong, although complex and conflicting	Populations might be growing more physically active regardless of community heart health interventions. Lack of statistically significant results might be due partly to design and measurement	We should examine the differences in the strategies used by those projects that found a positive effect and those that did not. More research is needed, especially on populations at high risk for low physical activity
Fogelholm & Lahti-Koski, 2002 [16]	The results on physical activity were positive in most studies but the effects on body weight were disappointing	There was a lack of evidence on important subgroups. Potential subgroup targets could be people of lower socioeconomic status, minority groups and older adults	No conclusion	Secular trends in healthier dietary choices and smoking cessation could dilute and confound effects. Lack of clear intervention effects could be due to methodological problems or to the cardiovascular disease focus of included studies	Future interventions should use components from previous interventions with a much stronger emphasis on physical and social environment. Also need national legislative policies
Jackson *et al.*, 2005a [15]	Lack of evidence	Lack of evidence	No evidence found	Not possible due to lack of evidence	Future research in this area should be rigorously designed and evaluated. Many recommendations made
Jackson *et al.*, healthy behavior change [14]	Lack of evidence	Lack of evidence	No evidence found	Not possible due to lack of evidence	Future research in this area should be rigorously designed and evaluated. Many recommendations made
King, 1998 [17]	Author’s position not clear. States that community coalition is a useful first step and physical activity should be made a focal point for intervention	Mass media interventions may need to target specific subgroups	No conclusion	Authors position not clear	The most exciting future prospect is social and environmental interventions
Murphy and Bauman, 2007 [13]	Much rhetoric but limited evidence of effectiveness of mass sporting events to increase physical activity	No conclusions	Few quality intervention evaluations in this field	Lack of evidence might be due to lack of coordination between organizers of mass sporting events and public health agencies and there are methodological difficulties which may make impacts hard to measure	Future mass events should include integrated and multi-sectoral physical activity and related planning and commit to investment in research for a better evidence base
Pate, 2000 [18]	Community based strategies to promote proper diet and physical activity ‘somewhat effective’ at improving physical activity behavior	No conclusions	There are many gaps in evidence	Author’s position not clear. Implies that better research would demonstrate effectiveness	More research is needed, especially work that links community-based initiatives to school-based interventions […] and strategies to involve multiple segments of the community. Makes detailed research recommendations
Sharpe, 2003 [19]	Author’s position not clear. Interventions have ‘value’ in promoting physical activity for arthritis and related disability. Important to create a supportive community environment with safe, accessible, and pleasant options	Market segmentation and tailoring to subgroups is essential (subgroups by location, ethnicity, income, age, sex, health status)	No conclusions	Author’s position not clear. Implies that there are differences between short and long term effects and enhanced by good assessment of community needs	Need for creating a linkage between successful person-focused, community-based programs for persons with arthritis and other broader- interventions targeting environmental and policy issues

### To what extent do reviews answering a similar research question cite other reviews on the same topic?

There was little citation of the eight reviews by one another. Only three reviews [16,18,19] cited any other of the reviews. Of a possible 28 instances where the eight reviews could have cited one other (once date of publication had been taken into account), there were only four instances of citation (Table 7). The four instances of citation were of the same two non-systematic reviews, one of which was cited by three different reviews (Table 7) [17,18].

**Table 7:** To what extent do the included reviews reference each other

	**King, 1998[**[17]**]**	**Dobbins & Beyers, 1999[**[12]**]**	**Pate, 2000[**[18]**]**	**Fogelholm & Lahti-Koski, 2002[**[16]**]**	**Sharpe, 2003[**[19]**]**	**Jackson*et al.*, 2005a[**[14]**]**	**Jackson*et al.*, 2005b[**[15]**]**	**Murphy & Bauman, 2007[**[13]**]**	**Total cited/total possible**
King, 1998	This was the first review of the eight to be published so we would not expect it to cite any of the others.								0/0
Dobbins & Beyers, 1999	-	-	-	-	-	-	-	-	0/0
Pate, 2000	Yes	-	-	-	-	-	-	-	1/1
Fogelholm & Lahti-Koski, 2002	Yes	No	No	-	-	No	No	-	1/5
Sharpe, 2003	Yes	No	Yes	-	-	-	-	-	2/3
Jackson *et al*., 2005a	No	No	No	No	No	-	No	-	0/6
Jackson *et al.*, 2005b	No	No	No	No	No	No	-	-	0/6
Murphy and Bauman, 2007	No	No	No	No	No	No	No	-	0/7
Total citations of review (column total)	3	0	1	0	0	0	0	0	4/28
									

- Indicates where we would not expect the review to be cited, either because it would be citing itself or because there is a year or less between review publication dates.

### Does the methodological quality of reviews answering a similar research question help us to understand any differences between included studies, results, and conclusions?

We found that the methodological quality of the reviews varied (Table 1). There were three ‘systematic reviews’ (Table 1) [12,14,15]. Only the two Cochrane reviews [14,15] met our criteria for an adequate search strategy. However, the searches by one other review met all search criteria except reporting that it searched using both free text and thesaurus terms. This can also be thought of as a ‘systematic’ review [12].

For the three systematic reviews (two of which were ‘empty’ reviews; that is, they did not contain any included primary studies), it was possible to explain why all non-included primary studies were not included [12,14,15]. However, in the lower quality reviews, it was more difficult to explain reasons for exclusion and almost half the exclusions in one such review could not be explained (*n* = 12/26 not explained; 46%) [17].

As two of the three ‘systematic’ reviews were ‘empty’, we could not meaningfully compare differences between included studies, results and conclusions in systematic and non-systematic reviews.

## Discussion

### Main findings

It was often difficult to identify ‘included’ studies and much deduction was needed in explaining why some primary studies may not have been included in a specific review.

We found little overlap of included studies within the eight reviews, despite the similarity of the research question. Studies with multiple publications were more likely to be included in reviews than shorter term studies which generated single publications. The results of studies with multiple publications were also more likely to be reported differently by different review authors.

Although search strategies in the majority of cases did not meet our quality threshold, the inclusion criteria of the reviews appeared to justify the lack of inclusion of specific primary studies. Unsurprisingly, it was easier to explain the exclusion of studies in better quality reviews, as they had clearer inclusion criteria and search strategies.

Reviews of longitudinal and multi-stage interventions were more likely to find larger studies, but less likely to report their findings comprehensively because these are dispersed across many publications, not all of which were necessarily reported.

Discrepancies in findings did not lead to discrepancies in conclusions. This may be because it is particularly challenging to show an impact arising from complex interventions and reviewers tended to be cautious with their interpretations.

There was little cross-citation between reviews and only the lower quality reviews cited other reviews in our analysis.

It was possible to explain why all non-included studies were absent from the systematic reviews, but more difficult to do so for the non-systematic reviews. (Since two out of the three systematic reviews were ‘empty’ we were unable to compare differences in terms of how reviews of different quality treated their included studies.)

### Strengths of this study

There are several strengths of this study. First, our searches were far-reaching and sensitive and our definition of ‘community intervention’ was broad. Consequently, the eight reviews analyzed here are likely to represent fully the group of reviews available at the time of the searches which aim to evaluate the effectiveness of community interventions for promoting physical activity. Secondly, by excluding reviews which were mainly descriptive, which did not draw conclusions specifically about physical activity or which restricted their population of interest, we ensured that the scope of the reviews was similar enough to warrant a comparison. Thirdly, we assessed the quality of the reviews and were able to comment on the relationship between review quality and our findings. It was necessary to use high levels of researcher judgment at several key stages of analysis: when classifying primary studies as ‘included’, when extracting authors’ conclusions and when assessing whether exclusion of primary data could be ascertained. We implemented quality assurance measures to minimize the potential for inconsistencies when extracting and analyzing data, especially for the lower-quality reviews which had less defined boundaries.

### Weaknesses of this study

Our analyses of reasons for exclusion of primary studies were based on the abstract of the included studies. It is possible that our analyses of the reasons for exclusion would have been different had we used the full text of the included studies and/or had contacted the review authors for data. We assumed that a primary study had been found and excluded by a review if we could justify its exclusion by the inclusion criteria or search/publication date. We cannot quantify how much primary data were never found by the reviews and cannot, therefore, comment on whether it is the scope of the review or the methods used that led to the lack of inclusion of specific primary studies.

We also acknowledge that since the searches for the original review of reviews were carried out in November/December 2007, other reviews on this topic have been published. These may reflect developments in review method that overcome some of the weaknesses in the reviewed evidence base; however, the general messages contained in this paper about understanding how different reviews on the same subject relate to one another will remain important to understand.

### Methodological issues

To some extent, we were surprised by our findings. We had expected to find greater overlap between reviews and, where overlap was limited, diversity in findings. The similarity in findings can be explained by the fact that no reviews found compelling evidence of effectiveness in the studies they included; they were all therefore cautious in their conclusions. This finding echoes the results of a similar study, that, even though the scope and quality assessment methods employed in health promotion reviews differed, this is ‘unlikely to divide opinion radically about effectiveness amongst cautious reviewers’ [64]. In contrast, two reviews with a similar research question came to very different conclusions about the effectiveness of interventions for childhood obesity [65]. In these reviews, conclusions were based on the results of randomized controlled trials (RCTs) and it may be that reviewers tend to be more cautious, and therefore their conclusions less divergent, when interpreting observational data.

The lack of overlap of primary studies warrants further examination, because it cannot be explained (entirely) in terms of deficiencies in the search strategies of the reviews, but rather seems to be due to differences in the scope (inclusion criteria) of the reviews which relates to heterogeneity in their review questions. This finding is consistent with other methodological studies that found that many apparent inconsistencies in the citation and selection of primary studies, especially non-RCTs, could be attributed to differences in inclusion criteria and outcome assessments of the reviews (rather than being due primarily to problems in their search strategies) [65,66]. Even though we had determined our sample of reviews to be as similar as possible in scope so that we could investigate overlap, in practice, the scope of the reviews did not overlap very much. This has important implications for the utility of reviews to inform policy and practice.

First, in areas where evaluation and impact measurement is known to be difficult and where research and policy interest is relatively recent, it is likely that the findings of reviews will reflect uncertainties in the primary studies and be less enlightening about the substantive topic. Review conclusions can only ever be as good as the available data on the topic [67]; this was certainly the case in the reviews that we examined. Across the topic of community interventions to promote physical activity, reviews were necessarily cautious in their findings because of uncertainties in the evidence base. While this is useful for researchers and research commissioners to know, it is less useful for people involved in determining policy and practice.

Second, dealing with linked publications (multiple publications from the same study) was complicated and confusing, both for ourselves and seemingly for the reviewers of our eight included reviews. To improve fidelity of reporting and ensure that all relevant evidence informs review results and conclusions, it is important to identify all publications from studies with multiple or staged evaluations. We therefore recommend that study authors aid researchers by clearly citing all previous and intended work in each publication and that this is also something that editors check before publication. Larger studies might consider keeping a website for the study which details all related publications (as some already do). Reviewers can search for multiple publications from a study by searching for papers by authors, studies and research groups that feature in the provisional list of included studies for the review. In order to build on existing knowledge, review authors should search for existing relevant reviews in the area and use this knowledge to contextualize their aims and findings. Inclusion (and citation) of relevant reviews will also help direct readers to relevant resources.

The study has also highlighted some of the unavoidable complexities that face potential users of systematic reviews. We placed ourselves in a hypothetical situation, but one that is similar to that faced by many policymakers and practitioners who would like their decisions to be informed by evidence; for example, a newly formed Health and Wellbeing board in the UK, tasked with reducing obesity among young people, might well want to examine what works in terms of promoting physical activity. If they used the map of community interventions and identified these eight reviews as being relevant, they would find that: while all the reviews were about the promotion of physical activity, they each had a particular ‘angle’, which determined the range of research they included; where the same studies were included in reviews, their findings were not always reported consistently; the concept of ‘community’ was often discussed in reviews, but there were also differences in its conceptualization; and on the whole, the reviews did not position themselves as contributing to a wider evidence base around the promotion of physical activity (as evidenced by the lack of inter-citation between them).

There was an inevitable tension in this analysis between a narrowness that ensured that all reviews were on exactly the same topic, and a breadth that ensured all potentially relevant reviews were included; the same tension concerning homogeneity of focus as exists in many systematic reviews in public health. Given that most public health decisions are about identifying solutions to a problem (in this case, increasing levels of physical activity), obtaining a range of reviews is to be expected; and the question that this paper begins to unpick emerges: ‘how coherent is the picture that emerges?’

Reviews which give a limited ‘slice’ of the evidence are extremely valuable if the policy/practice question is closely aligned to the scope of the review, but are less useful if they give only a partial picture. In our topic area however, even with the findings of all eight reviews at our disposal, we would not be confident that we were building on the results of all research about community interventions to promote physical activity, because each review contains a limited portion of the evidence and there may well be relevant studies that fall outside the scope of any of our reviews. (We should reiterate the point made above, that systematic review methods are developing quickly, and that some of these ‘gaps’ may now be filled.)

The above points relate to wider and unsolved issues about the amount of ‘work done’ in a review [68]. Some reviews have a relatively narrow focus, undertaking a detailed look at a relatively small area; there is additional ‘work’ to be done by users in identifying a range of such reviews and ‘synthesizing’ them to inform their particular decision. Other reviews are broader in scope which means that, potentially, less ‘work’ needs to be done by their users, though there is a tension between achieving both breadth and depth in the same review the risk being that broad reviews may suffer from a lack of focus and be deficient in essential detail [16]. While a detailed discussion of these issues is beyond the scope of this paper, we have highlighted areas within which review authors might usefully assist potential users.

## Conclusions

One possible future way forward is to undertake more systematic ‘maps’ of research activity. Systematic maps find and describe the research on a given topic and help researchers and policymakers to judge where there is and is not sufficient data to justify a narrow and in-depth review which seeks to answer a specific policy or practice question [32]. It is important, however, that systematic maps are kept updated and that funders allocated resources to this end. To maximize access to the knowledge gathered in systematic maps, they should be made freely available to researchers, funders, and policymakers.

Finally, we recommend for further reading the Guidelines for systematic reviews of health promotion and public health interventions [69] that was written by members of the Cochrane Public Health Review Group. This document discusses many of the issues mentioned above and aims to build reviewing capacity among those working in the difficult areas that create a great deal of the complexity identified in this analysis. Also, for those interested in the substantive topic of the reviews discussed here, we refer readers to a recent Cochrane review on the subject [70].

## Competing interests

The authors declare that they have no competing interests.

## Authors’ contributions

JW contributed to the design of the study, carried out all analyses, interpreted results, developed conclusions, and wrote the paper. JT conceived and designed the study, interpreted results, developed conclusions, and oversaw the writing of the paper. KD carried out quality assurance measures and commented on the writing of the paper. The content of the paper has been approved by all authors. All authors read and approved the final manuscript.
